# Changes in the geographical distribution of plant species and climatic variables on the West Cornwall peninsula (South West UK)

**DOI:** 10.1371/journal.pone.0191021

**Published:** 2018-02-05

**Authors:** Aleksandra Kosanic, Karen Anderson, Stephan Harrison, Thea Turkington, Jonathan Bennie

**Affiliations:** 1 University of Exeter, Centre for Geography Environment and Society, Penryn Campus, Penryn, United Kingdom; 2 University of Exeter, Environment and Sustainability Institute, Penryn Campus, Penryn, United Kingdom; 3 Centre for Climate Research Singapore, Meteorological Services Singapore, Singapore, Singapore; 4 Ecology Lab (Biology Department), University of Konstanz, Konstanz, Germany; Consejo Superior de Investigaciones Cientificas, SPAIN

## Abstract

Recent climate change has had a major impact on biodiversity and has altered the geographical distribution of vascular plant species. This trend is visible globally; however, more local and regional scale research is needed to improve understanding of the patterns of change and to develop appropriate conservation strategies that can minimise cultural, health, and economic losses at finer scales. Here we describe a method to manually geo-reference botanical records from a historical herbarium to track changes in the geographical distributions of plant species in West Cornwall (South West England) using both historical (pre-1900) and contemporary (post-1900) distribution records. We also assess the use of Ellenberg and climate indicator values as markers of responses to climate and environmental change. Using these techniques we detect a loss in 19 plant species, with 6 species losing more than 50% of their previous range. Statistical analysis showed that Ellenberg (light, moisture, nitrogen) and climate indicator values (mean January temperature, mean July temperature and mean precipitation) could be used as environmental change indicators. Significantly higher percentages of area lost were detected in species with lower January temperatures, July temperatures, light, and nitrogen values, as well as higher annual precipitation and moisture values. This study highlights the importance of historical records in examining the changes in plant species’ geographical distributions. We present a method for manual geo-referencing of such records, and demonstrate how using Ellenberg and climate indicator values as environmental and climate change indicators can contribute towards directing appropriate conservation strategies.

## Introduction

Recent climate change has become one of the main drivers of shifts in the geographical distributions of plant species [1, 2]. There are several ways in which species can respond to climate change: adapt, move in different directions in order to track suitable climates, (i.e. towards higher latitudes and elevations, or to the east and west) [3, 4], and go extinct locally, regionally, or, in a worst case scenario, globally [5–8]. Projections show that the increase of the global mean temperature by the year 2100 is very likely to be between 1.5°C and 4.0°C (depending on emission scenarios) and the impact on ecosystems will be unprecedented [9, 10]. Responses of plant species will depend on their genetic diversity and ability to adapt to the magnitude and rate of climate change, as well as availability of space for species to move into local microclimates [11–13]. Nevertheless, it has been shown that not all plant species will be equally sensitive to climatic change [2, 8, 14]. However, temperature is not the only aspect of the climate that is changing. Precipitation and the frequency of extreme events will also have an impact on vegetation, and the rate and magnitude of these climatic effects will differ regionally and locally [10, 15–17]. Changes in geographical distributions of vegetation at the local and regional scales can impact community composition, ecosystem function, and genetic diversity, which can make plants even more vulnerable to on-going environmental change [18–21]. Furthermore, changes in the distribution of vegetation on such scales could also affect regional identity and ecosystem services provision [22–24]. Therefore, there is a need for more research to focus on vegetation responses to climate change at local and regional scales [25, 26], particularly in order to identify vulnerable plant species. This can help to secure *in situ* management and prevent regional and local vegetation loss that could drive economic, social and environmental losses [2, 27]. Attribution of vegetation responses to climate change at local and regional scales, and identification of vulnerable species is nonetheless a difficult challenge in ecology; mainly because decadal patterns could be related to non-climatic factors, not least factors such as land use change [1, 28]. Hence, we need a better understanding of species’ individualistic responses to environmental change [8].

To track species’ responses to climate or environmental change, historical records (e.g. herbarium collections) represent exceptional sources of scientific and conservation data because they offer a means of tracking changes in species’ geographical distributions over time. So far, historical vegetation records have been used to analyse climatic effects on vegetation phenology [29–32], to predict changes in species’ distributions, to analyse patterns of plant species invasion, and to identify threatened species [33–35]; however, there is still a lack of regional and local scale studies. The major reason for the limited number of studies at the regional and local scales is not only a lack of multiple historical records (e.g. climatic and vegetation data) but also the lack of historical vegetation records in a precise geo-referenced form (i.e. geographical latitude and longitude) [36–38]. Therefore, a major task for scientists, museums, and archives is to deal with location uncertainties of historical records and to make such databases available in an accurate geo-referenced form [39–42]. This need has led to the design of web-based automated or semi-automated mapping applications such as BioGeomancer, MaNIS, MaPSTeDI [40, 43]; however, these applications are not available for all countries or regions, and in such instances geo-referencing needs to be performed manually. Manual geo-referencing, particularly on a specimen by specimen basis, is generally avoided as it has been characterised as time consuming [40, 44] and lacking detailed methodological guidance in the literature.

In this manuscript we present a method describing the process of manual geo-referencing of historical records (i.e. herbarium collections), in order to examine changes in the geographical distribution of plant species, using West Cornwall (South West England) as a study site with good availability of historical vegetation records [24]. We also assess whether Ellenberg values (EV) and climate indicator values (CV), both developed to characterise plant species ecology (i.e. species’ individual sensitivity to abiotic change) [45–47], can be used as tools to track climate and environmental change, and to show which species will be more sensitive to the change. For example, EV have been used previously to detect environmental change [48–50] and to document habitat quality [51]; however, there have been few studies examining if EV reflect a local or regional climate change signal [52–54]. Additionally EV can serve as a tool for detecting those plant species that are most vulnerable to climate change [52] and, potentially, for informing successful conservation strategies at local and regional scales. This study aims to address two key questions: 1) Can historical (herbarium) plant species data be used to evaluate changes in geographical distribution? 2) Is there a correlation between EV and CV of plant species and their distribution patterns?

## Data and methodology

### Contemporary plant species data (post-1900)

Contemporary spatial records (referred to in this paper as “post-1900”) of plant distribution in West Cornwall (South West England) were obtained from the online “Vascular Plants Database” of the National Biodiversity Network (NBN) [55]. The NBN database contains the distributions of 6669 taxa of flowering plants and ferns and contains mostly records from the “New Atlas of the British and Irish Flora” [56] and records collected by volunteer members of the Botanical Society of the British Isles (BSBI). NBN Vascular Plant records were validated by BSBI members and obtained at a 10x10 km grid resolution for this study.

### Historical plant species data (pre-1900)

**Fig 1:**
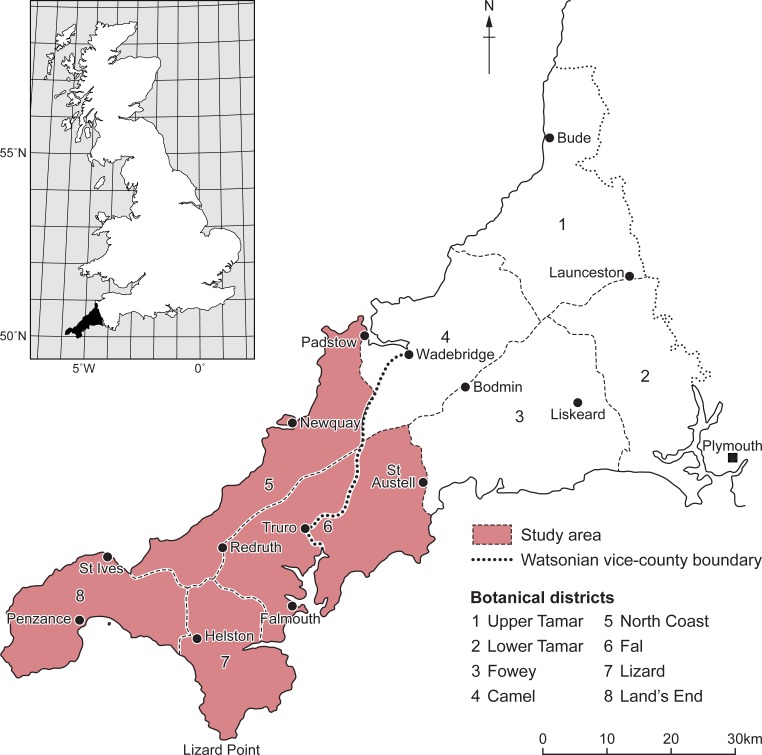
Botanical districts of historical herbarium data in Cornwall. Showing study area and botanical districts that were used in this research. Figure adapted from Davey [58].

Cornwall has a long history of botanical records that date back to Victorian times, which was encouraged by Natural History Societies at the time, in order to construct regional scientific knowledge [57]. In this study, historical records (referred to as “pre-1900”) were used from “The Flora of Cornwall” [58], a collection of all known herbarium data in the county of Cornwall and the Scilly Isles from the 18^th^ and 19^th^ centuries. In these records Cornwall is divided into eight botanical districts based on river basins [58]. Geo-referencing was undertaken for the 5^th^, 6^th^, 7^th^ and 8^th^ districts that cover the area of West Cornwall (Fig 1). Records contained both native and non-native species. Such historical data contain textual descriptions of localities where plant specimens were found (e.g. “*Achillea ptarmica*, first record: 1769; district 7: Porkellis Moor, Wendron, Coverack, *Emyln*” page 247), [58], rather than explicit definitions of longitude and latitude. We therefore acknowledge several uncertainties in the geo-referencing process: taxonomical inaccuracy; spatial error; bias associated with frequency; and time spent on data collection (i.e. some areas or species that could be poorly sampled). The latter uncertainty provides particular challenges [36] and details of our methods for dealing with these uncertainties are given below.

### Handling and spatial analysis of plant species records in ArcGIS

Manual geo-referencing of historical plant species data posed a methodological challenge [40] due to textual descriptions of specimen localities. Specifically, “The Flora of Cornwall” [58] contains descriptions of species (genus and specific epithet) and textual descriptions of geographic localities (i.e. places where specimens had been collected). In order to manually geo-reference these data and import into GIS software (ArcGIS) for subsequent spatial and temporal analysis, the following three steps were undertaken:

1) As it would be impractical to geo-reference all plant specimens in West Cornwall as recorded in the Flora of Cornwall [58], we created a baseline dataset using the “New Atlas of the British and Irish Flora” [56]. For this baseline dataset, 380 plant species were selected following two rules: *a)* they were detected in Cornwall pre-1970 by Preston et al. [56] and *b)* their geographical distribution (calculated as a change in areal extent) increased or decreased by more than 50% in the period from 1970 to 2002, also by Preston et al [56]. An electronic database was constructed from these data.

2) Species were then searched for in the herbarium collection “The Flora of Cornwall” [58], and those specimens found to be recorded in West Cornwall pre-1900 were geo-referenced as accurately as possible using ArcGIS. We used Google Earth at the initial stage of geo-referencing process to confirm textually described localities. Google Earth has been previously used in the geo-referencing process of historic herbaria and proved to be a useful tool as it allows quick detection of plant species localities from textual descriptions [44]. The accuracy of geo-referenced locations was cross-checked using online Ordnance survey archive maps for West Cornwall at a scale of 1:2500 [59]. Specimens that were found to have very ambiguous locality descriptions (e.g. “West Penwith area”) were excluded from the study.

Species without published Ellenberg values (see below) [47], synonyms, or species with incorrect taxonomy were also excluded from the database and subsequent analysis, following suggestions of Lavoie [60]. Taxonomic inaccuracy and possible synonyms were checked in The Plant List, the most extensive online database of all known plant species [61]. In total, 1187 plant specimens (comprising 120 plant species) from West Cornwall were included in the final spatial analysis database.

**Fig 2:**
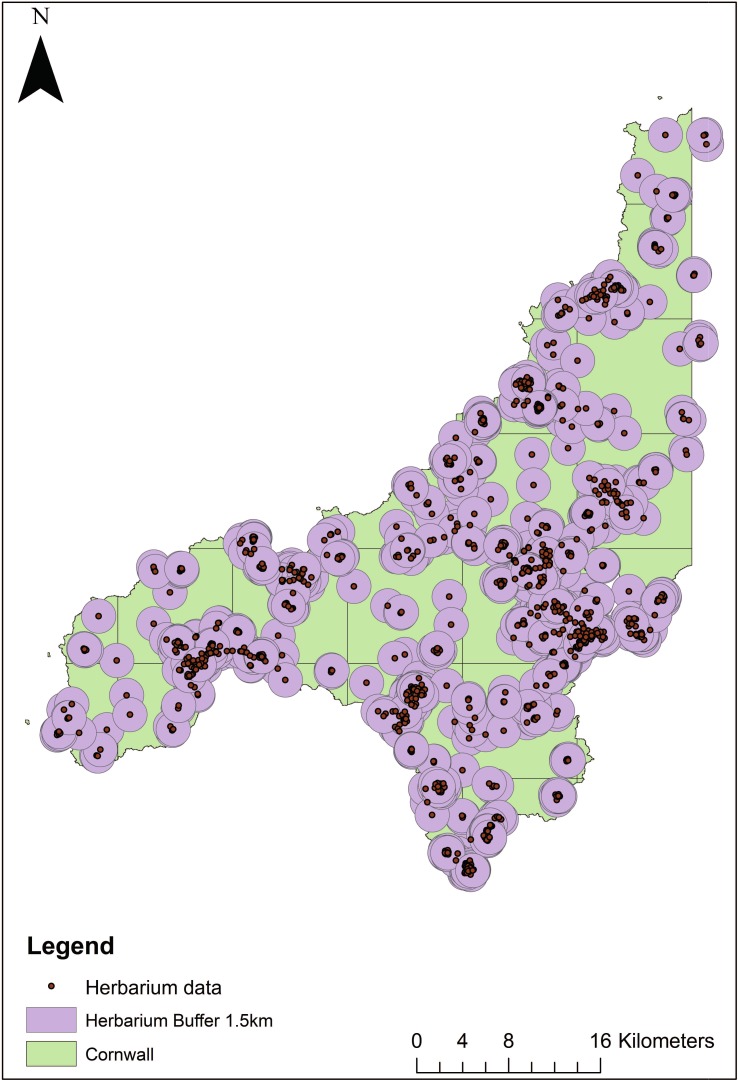
Geo-referencing of pre-1900 plant species data. Showing geo-referenced herbarium data with 1.5 km uncertainty buffers [66].

3) Information on specimen localities was imported from Google Earth into ArcGIS to complete the geo-referencing process and create distributional maps for the pre-1900 dataset. The extent of spatiotemporal uncertainty was then determined to create uncertainty ‘buffers’ for the pre-1900 data, which were applied to every record. This was done using a point radius method developed by Wieczorek et al. [39], which has been shown to be reliable [62, 63] when used as a part of automated or semi-automated geo-referencing programs. In this instance of manual geo-referencing, however, the point radius method was adapted as it would be impractical and time consuming to create an individual uncertainty buffer for each of the 1187 manually geo-referenced specimens. To determine a suitable radius for the buffers, we followed guidelines by Wieczorek et al. [64]: (1) for the ‘named places with a bounded area’ (e.g. towns or farms) we measured a maximum distance from the centre of a species’ named place to its furthest extent border; (2) for specimens between two ‘named places’ the buffer was calculated as half the distance between the centres of both named places; (3) for the specimens with a locality within ‘named places with undefined areas’ (i.e. places without a clear spatial boundary), for the extent measure we used half of the distance from the specimens’ locality coordinates to the centre of the nearest named place. Some specimens from Davey’s herbarium collection [58] had the names of geographic features as a location (e.g. Kennall river) or ‘offset localities’ with direction only, and without recorded distance or vice versa (e.g. North of Falmouth or 5 miles from Falmouth). Therefore, to create uncertainty buffers for all geo-referenced specimens we chose 50 random geo-referenced species’ localities (with bounded or undefined areas, and localities between the places), and their extent was calculated using the *Ruler* tool in Google Earth and *Measure* tool in ArcGIS. The upper quartile of all 50 extent distances was calculated to be 1.5 km, which was then applied as the uncertainty buffer around each specimen within ArcGIS (Fig 2). As historical maps from the same period as specimen collections were not available in digitised form, and clearly the extent of places have changed throughout history [39, 44], our calculated uncertainty buffers are most likely an overestimate rather than an underestimate.

**Fig 3:**
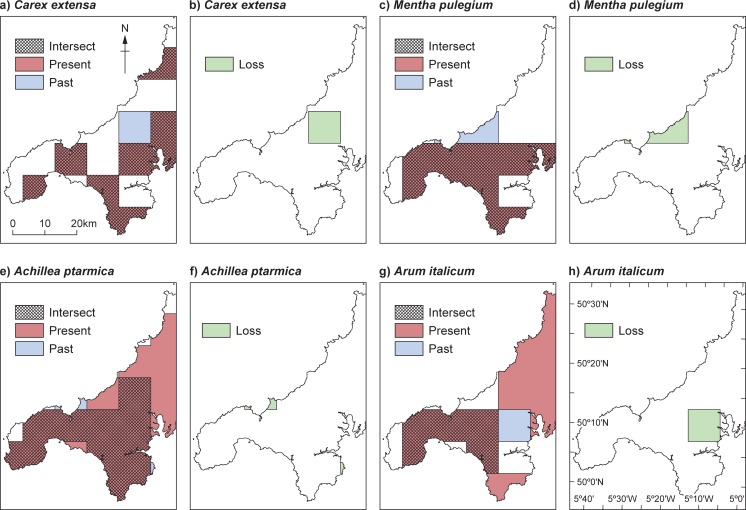
Example of pre-1900 and post-1900 changes in geographical distributions of plant species in West Cornwall. Showing present (only present) and past (only past) geographical distributions, intersect (showing overlap of present and past distributions), and loss for: a) Intersect *Carex extensa* b) Loss for *Carex extensa*; c) Intersect for *Mentha pulegium*; d) Loss for *Mentha pulegium*; e) Intersect for *Achillea ptarmica*; f) Loss for *Achillea ptarmica [66]*.

Upon creation, the uncertainty buffers in ArcGIS were attributed to the 10 km grid cells in West Cornwall using the *Spatial join* tool [65]. Contemporary data (post-1900) were also imported to ArcGIS and spatially joined to the 10 km grid cells. Both datasets were clipped to the shapefile of West Cornwall. Furthermore, for both datasets, polygons of species’ geographical distributions were then created using the *Dissolve* tool allowing the subsequent calculation of area loss. This step was necessary due to the geographical nature of West Cornwall as a peninsula, resulting in a proportion of many grid cells being taken up by ocean, and therefore analysis on a grid cell basis could create additional bias. To characterise changes in geographical coverage of plant species between pre-1900 and post-1900, spatial analysis was performed using the *Intersect* tool to identify species overlap. The local range loss of species between two the periods was also calculated using the *Symmetrical difference* tool (Fig 3), and actual loss was calculated by the function *Calculate geometry* in ArcGIS [65]. The difference in area covered in post-1900 records as a proportion of the original territory (i.e. species that occupied West Cornwall pre-1900) was also calculated.

### Analysis of plant species EV and CV and geographical distribution change

Ellenberg values were developed for each individual plant species in Central Europe by Ellenberg et al. [45] based on field observations showing plant species’ sensitivity to abiotic factors such as T-temperature, L-light, M-moisture of soil, R-reaction, S-salt concentration, K-continentality and N-nitrogen (soil fertility) [67, 68]. Each factor is measured on a nine to twelve rank scale depending on the region that they were calculated for [46]. Ellenberg values are related to a species’ synecological optimum (species interactions with the environment) rather than ecological ones [52]. The values used in this study were calibrated for UK plant species and scaled between 1–9 or 1–12 for each species (e.g. M = 1 indicates extreme dryness whereas M = 12 indicates almost constant submersion) [46, 47]. Hill et al. [47] omitted the calibration of the original EV for K-continentality and T-temperature as they were not applicable for the UK oceanic climate. Therefore, here we focus on EV for light (L), moisture (M) and nitrogen (N). Furthermore, instead of K-continentality and T-temperature EV, we used three CV from previously derived mean climatic data for the species range within 10 km grid cells for the British Isles [47]: (i) mean January temperature (Tjan), (ii) mean July temperature (Tjul), and (iii) mean precipitation (RR). To match the ordinal values of the three EV, temperature and precipitation indicators (Tjan, Tjul, and RR) were subdivided based on the values in Table 1, with lower values indicating the coldest/driest conditions and higher values indicating the warmest/wettest conditions (see S1 Table). The subdivisions were selected to have an even spread of species between the indicator values, while maintaining regular spacing and minimising the number of species more than 0.5°C and 100mm from the extreme indicator threshold values for temperature and precipitation CV, respectively. The maximum, minimum and median mean temperature and precipitation values are in Table 2.

**Table 1:** Classifications for three climate indicator values (CV): Mean January temperature (Tjan), mean July temperature (Tjul), and mean precipitation (RR).

Indicator value	Tjan (°C)	Tjul (°C)	RR (mm)
1	<2.5	< 13.5	< 700
2	2.5–3.0	13.5–14.0	700–800
3	3.0–3.5	14.0–14.5	800–900
4	3.5–4.0	14.5–15.0	900–1000
5	4.0–4.5	15.0–15.5	1000–1100
6	4.5–5.0	15.5–16.0	1100–1200
7	5.0–5.5	16.0–16.5	1200–1300
8	≥5.5	≥16.5	≥1300

**Table 2:** The minimum, median and maximum value for each Ellenberg value (EV), light (L), moisture (M), nitrogen (N) and climate indictor value (CV), mean January temperature (Tjan), mean July temperature (Tjul), and mean precipitation (RR).

Variable	Minimum	Median	Maximum
(Tjan)	1.9°C	3.8°C	6.8°C
(Tjul)	13.0°C	15.6°C	16.6°C
(RR)	604mm	900mm	1483mm
(L)	4	7	9
(M)	2	5	12
(N)	1	5	9

Finally, the percentage of the pre-1900 area of each species that had no records in the post-1900 records was determined as a measure of area loss. We tested whether losses were more pronounced for species traits (EV and CV). The analysis compared each pair of indicator values, to detect any non-montonic relationships that could be missed by the Pearson’s correlation coefficient or Kendall’s Tau. The non-parametric Mann-Whitney U test was used to determine whether the percentage of area lost was statistically different (p value < 0.05) between two indicator values. The Mann-Whitney U test (also referred to as Mann-Whitney-Wilcoxon) has been used in other studies to determine the significant difference between two independent groups of data [69]. The test was necessary to test both EV and CV and determine which species with their associated values experienced a higher loss and were thus potentially more vulnerable to environmental change (see S1 Table). As an additional test, we also developed a Generalized Linear Model (GLM) in R-3.3.2 [70], in order to test the relation between percentage of area lost (response variable) and climatic values (explanatory variables) that were used in creating a substitute for the original EV [47]. The area lost were transformed using an arc-sine transformation since the data was proportional and bounded between zero and one.

## Results

### Spatial analysis of change in plant species geographical distribution

Of the 120-plant species analysed, spatial overlap between the pre-1900 and post-1900 datasets was found for 116 species, whereas 5 species appeared only in either post-1900 or pre-1900 datasets or without an intersect (Fig 3). A decrease in geographical extent was found for 19 species (the decrease was larger than 50% for 6 species), and no change in geographical distribution was found for 10 species. Species with the highest losses across West Cornwall are shown in Table 3.

**Table 3:** Species with the largest change in geographical distribution (in terms of the lost area) in West Cornwall.

Species	Km^2^
*Anagallis arvensis subsp*. *foemina*	484
*Anthemis cotula*	452
*Campanula rotundifolia*	325
*Clinopodium acinos*	929
*Cystopteris fragilis*	611
*Genista anglica*	217
*Juncus maritimus*	257
*Lavatera cretica*	210
*Linum usitatissimum*	212
*Medicago sativa subsp*. *falcata*	469
*Scilla autumnalis*	327

### Ellenberg values and climate indicator values

The ranges of CV and EV for the 120-species analysed are presented in Table 1B. For EV, Moisture (M) had the widest range (1–12) followed by Nitrogen (N) (1–9), and the narrowest range was for Light (L) (4–9). Based on the CV criteria, all three indicators span the same range (1–8).

**Fig 4:**
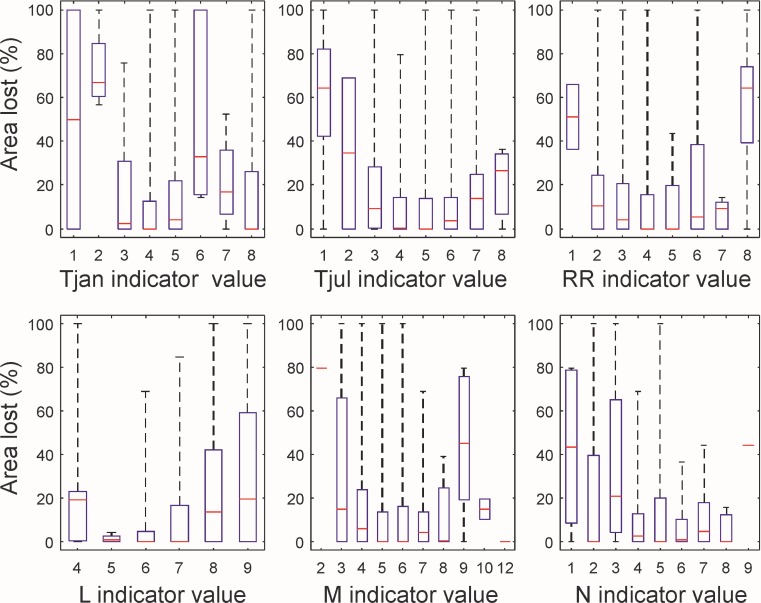
Pre-1900 area loss for Ellenberg values (EV) and climate indicator values (CV). Showing percentage of the pre-1900 area that was lost post-1900 for CV (top), and EV (bottom). Climate indicator values are ranked based on temperature (Tjan and Tjul) and precipitation (RR), with a value of 1 representing coldest/driest conditions and a value of 8 representing the warmest/wettest conditions. Three EV are included: light (L); moisture (M); and nitrogen (N). Median values shown in red and blue rectangles outline the 25^th^ to 75^th^ percentiles. EV relevant for only one species are shown as a single line (i.e. M = 2 and 12 and N = 9).

The percentage area losses between post- and pre-1900 calculated in relation to CV are shown in Fig 4 (top row). For Tjan, species with cold temperatures (Tjan = 2) had the highest percentage area loss, which was significantly greater than those with a value of 3, 4, 5, 7 and 8. Species with Tjan indicator values of 4 and 5 also showed a significantly lower reduction in extent than species for Tjan = 6 (Table 4). Therefore, except for Tjan = 6, species with colder winter temperatures generally show greater losses than those with warmer temperatures. For summer temperatures (Tjul), species with the lowest temperatures (Tjul = 1) lost a significantly higher percentage of the pre-1900 area than those with values of 4 and 5, with no significant differences between the other indicator values (Table 4). Based on the annual precipitation, species with RR = 8 showed significantly greater losses in area relative to pre-1900 than those with an indicator values of 2, 3, 4, 5, and 7. Species for which RR = 1 also had a higher median value of percentage area lost, but this was not significantly different to the other categories due to few species in this group (Table 4). Overall, CV indicate that species with cooler winter and summer temperatures, and higher annual rainfall had a greater percentage loss in area between pre- and post-1900.

**Table 4:** Mann-Whitney U test U and p values for pairs of environmental and climate indictor values (EV and CV, respectively). Only the significant results are shown (p value < 0.05).

CV	Indicator values		U	p value	EV	Indicator values		U	p value
Tjan	2	3	41.0	0.010	L	4	6	84.0	0.045
	2	4	216.5	0.002		6	8	359.5	0.009
	2	5	105.5	0.004		6	9	179.5	0.012
	2	7	16.0	0.029		7	8	981.5	0.008
	2	8	32.5	0.022		7	9	494.5	0.015
	3	6	55.0	0.025	M	5	9	135.5	0.015
	4	6	303.0	0.001		6	9	57.0	0.043
	5	6	139.0	0.011	N	1	6	108.0	0.032
Tjul	1	5	88.0	0.029		3	4	178.0	0.023
	1	6	111.5	0.034		3	5	412.0	0.011
RR	2	8	125.0	0.029		3	6	308.5	0.003
	3	8	203.5	0.005		3	7	190.0	0.027
	4	8	145.5	0.006		3	8	80.5	0.040
	5	8	86.0	0.005					
	7	8	31.0	0.030					

The GLM results are somewhat consistent with the findings above. Based on the GLM, the probability of losing species increases for species with higher RR values (Table 5). Furthermore, there are indications that the loss will be greater for species with lower January temperature and higher July temperature, however, these findings are short of statistical significance (Table 5).

**Table 5:** GLM analysis with area lost as response and climatic values as explanatory variable (p < 0.05).

	Estimate Std.	Error t	t value	Pr (>|t|)
(Intercept)	-5.84	3.43	-1.700	0.092
Tjan	-0.16	0.09	-1.704	0.091
Tjul	0.35	0.20	1.766	0.080
RR	0.002	0.001	2.031	0.045

The percentage of area lost in relation to EV is shown in Fig 4 (bottom row). Indicator values observed by only one species are shown as a single red line in Fig 4. For light (L) there was no clear pattern; losses for species with L = 4 were significantly higher than those with L = 6, while species with L = 8 or 9 showed significantly greater losses than those with L = 6 or 7 (Table 4). For moisture (M), excluding indicator values applicable to only one species (M = 2 and 12), species with moderate values (M = 4 to 8) had lower median losses than those with moderate-extreme values (M = 3, 9, 10) (Table 4). However, when considering all species for an indicator value, only species with M = 9 showed significantly greater losses than those with a value of 5 or 6 (Table 4). For nitrogen (N), species with lower indicator values had a higher percentage of area lost, with those having N = 1 showing significantly greater loss than those with N = 6, and those with N = 3 showing significantly greater loss than those with N = 4 to 8 (Table 4).

## Discussion

We have framed the discussion according to the two initial questions posed at the beginning of the manuscript:

1) Can historical (herbarium) plant species data be used to evaluate changes in geographical distribution?

Historical biodiversity collections are often associated with uncertainties and limitations [71], yet they still offer an enormous source of information on past geographical distributions, and their value is recognised in a context of evaluating present and future anthropogenic impacts on biodiversity [29, 72, 73]. Although such collections can be used in research, projections for future biodiversity responses, conservation purposes, and education [71, 74], most of the collections are still inaccessible as they are locked in a form of descriptive locality information. Recently museums and herbaria associations worldwide are making an enormous effort for: “*(i) the building*, *sharing*, *and preservation of digital collections; (ii) creation of tools (particularly*, *identification tools) and services; (iii) influencing and supporting innovation in communication between users; and (iv) the development of strategic partnerships for further digital library development”* [75] (page 44), however, surprisingly manual geo-referencing is often omitted as it has been perceived as time consuming, requires additional searching for resources such as archive maps or gazetteers [40, 76], and it poses a question of how to deal with the spatial uncertainty of textually-described localities manually.

In the past 15 years much more emphasis has been placed on automated and semi-automated geo-referencing tools [43], yet such tools are not the solution for all “locked” historical records as they are not applicable for all regions. Therefore, here we presented a method and demonstrated that historical records from regional and local herbarium collections can be manually geo-referenced with an assessment of spatial error, and integrated into a spatial assessment of distribution change across landscapes and can be used to understand potential drivers of that change. Still, one of the main criticisms of using historical plant records to track distributional change is that variation in collection methods could result in biased data [77–80]. Historical vegetation records were rarely collected systematically with equal effort across geographic space, so the absence of a record from locality does not mean that a species was absent. Therefore, we agree with Elith and Leathwick [74] that analysis of changes in species in geographical distributions using historical (e.g. herbarium) records should concentrate on loss and not gain. Furthermore, uncertainties in historical records could also be related to the quality of local and regional records, and a more cautious approach is needed if records are from regions where national biodiversity monitoring is scarce, affected by war or political instability, and regions with undeveloped transportation infrastructure [81, 82]. Nevertheless, in such regions even contemporary (i.e. 20^th^ and 21^st^ century) biodiversity records can be affected by collection bias [81] so we suggest that a detailed inspection of historical/contemporary biodiversity records (e.g. locality, date of collection, field notes) is required before assessing changes in local/regional distributions. To summarise, and answering the initial question directly, herbarium data can be used to evaluate vegetation change but users must acknowledge uncertainties in historical records to overcome a challenging process of manual geo-referencing.

2) Is there a correlation between EV and CV of plant species and their distribution patterns?

Only a few studies have looked at whether changes in geographical distributions of plant species and their associated Ellenberg indicator values follow regional climate variability [52, 83], and this has shown to be true to a certain extent due to microclimatic variations [84] showing we need more local and regional climate change analysis. Our results showed that species with colder average temperature Tjan values had a greater percentage loss of area than other species between pre-1900 and post-1900 datasets. These findings are consistent with results by Maclean et al. [26] who detected losses in the region (West Cornwall) for grassland species with low temperature requirements. The changes found in plant species geographical distributions and their associated CV also follow previous findings on climate change in the region [85]. For example, the results for plant species change and associated Tjul, are also consistent with previous results on climate variability in West Cornwall that show a positive trend in summer temperatures in the 20^th^ and 21^st^ centuries [85].

We found that changes in species’ geographical distributions correlated with rainfall (RR) and moisture (M) indicator values. Climate indicator values for RR and EV for M showed the greatest losses in pre-1900 area for species with the highest and lowest precipitation requirements, and for those with moderately extreme M values. GLM results also showed that the loss will be larger for the species with the higher RR values. Although these results do not follow the previous findings by Kosanic et al. [85], as no positive trends in annual or seasonal precipitation were detected, they are in line with Maclean et al. [26] who detected a shift in plant communities towards species with lower moisture requirements over the Lizard Peninsula. These results confirm high spatial variability of both temperature and precipitation effects, suggesting that more research on local vegetation response and microclimate is needed [13, 84, 86]. More local scale research will bring not only a clearer understanding of vegetation-climate change relationships but will also help to identify new microclimates that could buffer climate change effects and offer opportunities for targeted *in situ* conservation strategies [26, 87]. On the other hand, our result showing a smaller loss of moderately wetter species could also reflect land use changes, as specialist wetland or drought-tolerant species are expected to be lost as wetter and/or drier habitats become scarce or degraded. These results demonstrate that we need reliable information on local climate variability in the post-industrial era and that we need a better understanding of how plant species react to extreme climatic events.

Significant differences were also found for the non-climatic indicator values. A few significant differences were found for the light (L) EV showing a greater loss for specialist species (i.e. ones that require low light and high light environments), which may be linked to environmental change and with changes in species composition [49, 88].

Changes in the geographical distribution of plant species associated with nitrogen (N) showed a larger loss of area coverage for those with a lower N requirement. Higher nutrient availability as a result of changed and intensified agricultural practices may cause the prevalence of highly-competitive species and low-nutrient species are being out-competed [89]. Both changes in a plant species distributions with high or low L and N requirements could reflect a greater importance of non-climatic drivers such as changes in land use and increased urbanisation in the region during 20^th^ and 21^st^ centuries [90].

## Conclusion

This study demonstrates a novel method for incorporating spatial uncertainty in the manual geo-referencing of herbarium records and shows how to tackle the limitations of historical records [71]. We successfully use this approach to track changes in the geographical distributions of plant species at a local/regional scale. Historical records have a tremendous importance for analysing past changes in vegetation distribution that offer insight into responses to future as well as past environmental change. Our results show that the distributions of plant species with different EV and CV have also changed through time, and that they reflect the climatic variability of West Cornwall to some extent.

This approach can contribute towards identification of more sensitive and therefore more vulnerable plant species at the regional scale and should support more targeted *in situ* conservation strategies [27]. We argue that further research should be conducted on microclimates [13, 26, 84, 91], land use change, and species distribution changes, providing a firmer link between EV and CV, and changes in plant species geographical distributions. More research on EV and CV could lead not only towards clearer attribution of plant species’ responses to environmental change but also towards the detection of microrefugia sites and, therefore, could be used as a tool to preserve species in the region. To preserve species locally and regionally is important not only from the perspective of ecosystem services, regional identity, and human well-being, but also in a context of genetic diversity, an important component of species’ resilience in the face of future climate change [9, 24, 92, 93].

## Supporting information

S1 Table Plant species and climatic variables. Showing: plant species (ID), Area that was lost (%), Classified climate indicator values (CV), Ellenberg values (EV), Mean climatic data for the plant species (Raw CV).
(PDF)

## References

[1] Parmesan C, Duarte C, Poloczanska E, Richardson AJ, Singer MC. Overstretching attribution. Nature Clim Change. 2011;1(1):2–4.

[2] Pacifici M, Foden WB, Visconti P, Watson JEM, Butchart SHM, Kovacs KM, et al Assessing species vulnerability to climate change. Nature Clim Change. 2015;5(3):215–24. doi: 10.1038/nclimate2448

[3] Palmer G, Hill JK, Brereton TM, Brooks DR, Chapman JW, Fox R, et al Individualistic sensitivities and exposure to climate change explain variation in species’ distribution and abundance changes. Science Advances. 2015;1(9). doi: 10.1126/sciadv.1400220 26601276 10.1126/sciadv.1400220 PMC4646790

[4] Chen I-C, Hill JK, Ohlemüller R, Roy DB, Thomas CD. Rapid Range Shifts of Species Associated with High Levels of Climate Warming. Science. 2011;333(6045):1024–6. doi: 10.1126/science.1206432 21852500 10.1126/science.1206432

[5] Parmesan C. Ecological and Evolutionary Responses to Recent Climate Change. Annual Review of Ecology, Evolution, and Systematics. 2006;37(1):637–69. doi: 10.1146/annurev.ecolsys.37.091305.110100

[6] Cardinale BJ, Duffy JE, Gonzalez A, Hooper DU, Perrings C, Venail P, et al Biodiversity loss and its impact on humanity. Nature. 2012;486(7401):59–67. doi: 10.1038/nature11148 22678280 10.1038/nature11148

[7] Lenoir J, Gégout JC, Marquet PA, de Ruffray P, Brisse H. A Significant Upward Shift in Plant Species Optimum Elevation During the 20th Century. Science. 2008;320(5884):1768–71. doi: 10.1126/science.1156831 18583610 10.1126/science.1156831

[8] Parmesan C, Hanley ME. Plants and climate change: complexities and surprises. Annals of Botany. 2015;116(6):849–64. doi: 10.1093/aob/mcv169 26555281 10.1093/aob/mcv169 PMC4640131

[9] Bellard C, Bertelsmeier C, Leadley P, Thuiller W, Courchamp F. Impacts of climate change on the future of biodiversity. Ecology letters. 2012;15(4):365–77. doi: 10.1111/j.1461-0248.2011.01736.x 22257223 10.1111/j.1461-0248.2011.01736.x PMC3880584

[10] IPCC. Climate Change 2014, Synthesis Report, Summary for Policymakers. Contribution of Working Groups I, II and III to the Fifth Assessment Report of the Intergovernmental Panel on Climate Change. Geneva, Switzerland: 2014.

[11] Loarie SR, Duffy PB, Hamilton H, Asner GP, Field CB, Ackerly DD. The velocity of climate change. Nature. 2009;462(7276):1052–5. doi: 10.1038/nature08649 20033047 10.1038/nature08649

[12] Alsos IG, Ehrich D, Thuiller W, Eidesen PB, Tribsch A, Schönswetter P, et al Genetic consequences of climate change for northern plants. Proceedings of the Royal Society B: Biological Sciences. 2012;279(1735):2042–51. doi: 10.1098/rspb.2011.2363 22217725 10.1098/rspb.2011.2363 PMC3311896

[13] Suggitt AJ, Wilson RJ, August TA, Beale CM, Bennie JJ, Dordolo A, et al Climate change refugia for the flora and fauna of England. Sheffield: Natural England, 2014.

[14] Corlett RT, Westcott DA. Will plant movements keep up with climate change? Trends in Ecology & Evolution. 2013;28(8):482–8. 23721732 10.1016/j.tree.2013.04.003

[15] Zhao M, Running SW. Drought-Induced Reduction in Global Terrestrial Net Primary Production from 2000 Through 2009. Science. 2010;329(5994):940–3. doi: 10.1126/science.1192666 20724633 10.1126/science.1192666

[16] Coumou D, Rahmstorf S. A decade of weather extremes. Nature Clim Change. 2012;2(7):491–6.

[17] Min S-K, Zhang X, Zwiers FW, Hegerl GC. Human contribution to more-intense precipitation extremes. Nature. 2011;470(7334):378–81. doi: 10.1038/nature09763 21331039 10.1038/nature09763

[18] Richardson AD, Andy Black T, Ciais P, Delbart N, Friedl MA, Gobron N, et al Influence of spring and autumn phenological transitions on forest ecosystem productivity. Philosophical Transactions of the Royal Society of London B: Biological Sciences. 2010;365(1555):3227–46. doi: 10.1098/rstb.2010.0102 20819815 10.1098/rstb.2010.0102 PMC2981939

[19] Butchart SHM, Walpole M, Collen B, van Strien A, Scharlemann JPW, Almond REA, et al Global Biodiversity: Indicators of Recent Declines. Science. 2010;328(5982):1164–8. doi: 10.1126/science.1187512 20430971 10.1126/science.1187512

[20] Vilà M, Ibáñez I. Plant invasions in the landscape. Landscape Ecol. 2011;26(4):461–72. doi: 10.1007/s10980-011-9585-3

[21] Matthies D, Bräuer I, Maibom W, Tscharntke T. Population size and the risk of local extinction: empirical evidence from rare plants. Oikos. 2004;105(3):481–8. doi: 10.1111/j.0030-1299.2004.12800.x

[22] Balvanera P, Siddique I, Dee L, Paquette A, Isbell F, Gonzalez A, et al Linking Biodiversity and Ecosystem Services: Current Uncertainties and the Necessary Next Steps. BioScience. 2013:1–9. doi: 10.1093/biosci/bit003

[23] de Oliveira LEC, Berkes F. What value São Pedro's procession? Ecosystem services from local people's perceptions. Ecological Economics. 2014;107(0):114–21.

[24] Kosanic A, Anderson K, Frère CH, Harrison S. Regional vegetation change and implications for local conservation: An example from West Cornwall (United Kingdom). Global Ecology and Conservation. 2015;4:405–13.

[25] Keppel G, Van Niel KP, Wardell-Johnson GW, Yates CJ, Byrne M, Mucina L, et al Refugia: identifying and understanding safe havens for biodiversity under climate change. Global Ecology and Biogeography. 2012;21(4):393–404. doi: 10.1111/j.1466-8238.2011.00686.x

[26] Maclean IMD, Hopkins JJ, Bennie J, Lawson CR, Wilson RJ. Microclimates buffer the responses of plant communities to climate change. Global Ecology and Biogeography. 2015:n/a-n/a. doi: 10.1111/geb.12359

[27] Greenwood O, Mossman HL, Suggitt AJ, Curtis RJ, Maclean IMD. Review: Using in situ management to conserve biodiversity under climate change. Journal of Applied Ecology. 2016;53(3):885–94. doi: 10.1111/1365-2664.12602 27609987 10.1111/1365-2664.12602 PMC4991270

[28] Parmesan C, Yohe G. A globally coherent fingerprint of climate change impacts across natural systems. Nature. 2003;421(6918):37–42. doi: 10.1038/nature01286 12511946 10.1038/nature01286

[29] Calinger KM, Queenborough S, Curtis PS. Herbarium specimens reveal the footprint of climate change on flowering trends across north-central North America. Ecology letters. 2013;16(8):1037–44. doi: 10.1111/ele.12135. PMC3806244. 23786499 10.1111/ele.12135 PMC3806244

[30] Panchen ZA, Primack RB, Aniśko T, Lyons RE. Herbarium specimens, photographs, and field observations show Philadelphia area plants are responding to climate change. American Journal of Botany. 2012;99(4):751–6. doi: 10.3732/ajb.1100198 22447982 10.3732/ajb.1100198

[31] Gallagher RV, Hughes L, Leishman MR. Phenological trends among Australian alpine species: using herbarium records to identify climate-change indicators. Australian Journal of Botany. 2009;57(1):1–9. doi: 10.1071/BT08051

[32] Matthews ER, Mazer SJ. Historical changes in flowering phenology are governed by temperature × precipitation interactions in a widespread perennial herb in western North America. New Phytologist. 2016;210(1):157–67. doi: 10.1111/nph.13751 26595165 10.1111/nph.13751

[33] Lienert J, Fischer M, Diemer M. Local extinctions of the wetland specialist Swertia perennis L. (Gentianaceae) in Switzerland: a revisitation study based on herbarium records. Biological Conservation. 2002;103(1):65–76.

[34] Van den Eynden V, Oatham MP, Johnson W. How free access internet resources benefit biodiversity and conservation research: Trinidad and Tobago's endemic plants and their conservation status. Oryx. 2008;42(03):400–7. doi: 10.1017/S0030605308007321

[35] Rivers MC, Taylor L, Brummitt NA, Meagher TR, Roberts DL, Lughadha EN. How many herbarium specimens are needed to detect threatened species? Biological Conservation. 2011;144(10):2541–7. doi: 10.1016/j.biocon.2011.07.014

[36] Graham CH, Ferrier S, Huettman F, Moritz C, Peterson AT. New developments in museum-based informatics and applications in biodiversity analysis. Trends in Ecology & Evolution. 2004; 19(9):497–503. doi: 10.1016/j.tree.2004.07.006 16701313 10.1016/j.tree.2004.07.006

[37] Soberon J, Peterson T. Biodiversity informatics: managing and applying primary biodiversity data. Philosophical Transactions of the Royal Society of London Series B: Biological Sciences. 2004;359(1444):689–98. doi: 10.1098/rstb.2003.1439 15253354 10.1098/rstb.2003.1439 PMC1693343

[38] Feeley KJ, Silman MR. Modelling the responses of Andean and Amazonian plant species to climate change: the effects of georeferencing errors and the importance of data filtering. Journal of Biogeography. 2010;37(4):733–40. doi: 10.1111/j.1365-2699.2009.02240.x

[39] Wieczorek J, Guo Q, Hijmans R. The point-radius method for georeferencing locality descriptions and calculating associated uncertainty. International Journal of Geographical Information Science. 2004;18(8):745–67. doi: 10.1080/13658810412331280211

[40] Guralnick RP, Wieczorek J, Beaman R, Hijmans RJ, the BioGeomancer Working G. BioGeomancer: Automated Georeferencing to Map the World's Biodiversity Data. PLoS biology. 2006;4(11):e381 doi: 10.1371/journal.pbio.0040381 17105347 10.1371/journal.pbio.0040381 PMC1637066

[41] Graham CH, Elith J, Hijmans RJ, Guisan A, Townsend Peterson A, Loiselle BA, et al The influence of spatial errors in species occurrence data used in distribution models. Journal of Applied Ecology. 2008;45(1):239–47. doi: 10.1111/j.1365-2664.2007.01408.x

[42] Elith J, Graham C H., Anderson R P., Dudík M, Ferrier S, Guisan A, et al Novel methods improve prediction of species’ distributions from occurrence data. Ecography. 2006;29(2):129–51. doi: 10.1111/j.2006.0906–7590.04596.x

[43] Auer T, MacEachren AM, McCabe C, Pezanowski S, Stryker M. HerbariaViz: A web-based client–server interface for mapping and exploring flora observation data. Ecological Informatics. 2011;6(2):93–110.

[44] Garcia-Milagros E, Funk VA. data: Improving the use of information from museum specimens: Using Google Earth to georeference Guiana Shield specimens in the US National Herbarium. Frontiers of Biogeography. 2010;2(3).

[45] Ellenberg H, Weber HE, Düll R, Wirth V, Werner W, Paulißen D. Zeigerwerte von Pflanzen in Mitteleuropa (Indicator values for plants of Central Europe), Scripta Botanica 2001.

[46] Hill MO, Roy DB, Mountford JO, Bunce RGH. Extending Ellenberg's indicator values to a new area: an algorithmic approach. Journal of Applied Ecology. 2000;37(1):3–15. doi: 10.1046/j.1365-2664.2000.00466.x

[47] Hill MO, Preston CD, Roy DB. PLANTATT—attributes of British and Irish Plants: status, size, life history, geography and habitats. 2004.

[48] Diekmann M. Species indicator values as an important tool in applied plant ecology–a review. Basic and Applied Ecology. 2003;4(6):493–506.

[49] Dzwonko Z. Assessment of light and soil conditions in ancient and recent woodlands by Ellenberg indicator values. Journal of Applied Ecology. 2001;38(5):942–51. doi: 10.1046/j.1365-2664.2001.00649.x

[50] Häring T, Reger B, Ewald J, Hothorn T, Schröder B. Predicting Ellenberg's soil moisture indicator value in the Bavarian Alps using additive georegression. Applied Vegetation Science. 2012:n/a-n/a. doi: 10.1111/j.1654-109X.2012.01210.x

[51] Kollmann J, Fischer A. Vegetation as indicator for habitat quality. Basic and Applied Ecology. 2003;4(6):489–91.

[52] Pignatti S, Bianco P, Fanelli G, Guarino R, Petersen J, Tescarollo P. Reliability and effectiveness of Ellenberg’s indices in checking flora and vegetation changes induced by climatic variations In: Walther GR, Burga CA, Edwards PJ, editors. “Fingerprints” of Climate Change: Springer US; 2001 p. 281–304.

[53] Renetzeder C, Knoflacher M, Loibl W, Wrbka T. Are habitats of Austrian agricultural landscapes sensitive to climate change? Landscape and Urban Planning. 2010;98(3–4):150–9. doi: 10.1016/j.landurbplan.2010.08.022

[54] Van Der Veken S, Bossuyt B, Hermy M. Climate gradients explain changes in plant community composition of the forest understory: an extrapolation after climate warming. Belgian Journal of Botany. 2004;137(1):55–69.

[55] NBN. Vascular Plants Database and Vascular Plants Databese additions since 2000. Natural Biodiversity Network,; 2013; Data acess set to 10 km resolution. https://data.nbn.org.uk/. Accessed 16 June 2013.

[56] Preston CD, Pearman DA, Dines TD. New Atlas of the British and Irish Flora. 1st ed. Press OU, editor. Oxford: Oxford University Press; 2002.

[57] Naylor S. The field, the museum and the lecture hall: the spaces of natural history in Victorian Cornwall. Transactions of the Institute of British Geographers. 2002;27(4):494–513. doi: 10.1111/1475-5661.00067

[58] Davey HF. The Flora of Cornwall. 2nd ed. Wakefield, West Yorkshire, England: EP Publishing Limited; 1909.

[59] OS. Old-Maps.co.uk, 1:2500. 2010. https://www.old-maps.co.uk/#/Map/180500/32500. Accessed 10 April 2013.

[60] Lavoie C. Biological collections in an ever changing world: Herbaria as tools for biogeographical and environmental studies. Perspectives in Plant Ecology, Evolution and Systematics. 2013;15(1):68–76.

[61] The Plant List. A working list of all plant species. 2013; Version 1.1. http://www.theplantlist.org/. Accessed 9 January 2013.

[62] Doherty P, Guo Q, Liu Y, Wieczorek J, Doke J. Georeferencing Incidents from Locality Descriptions and its Applications: a Case Study from Yosemite National Park Search and Rescue. Transactions in GIS. 2011;15(6):775–93. doi: 10.1111/j.1467-9671.2011.01290.x

[63] Chapman AD, Wieczorek J. A guide to the best practices for georeferencing biological species written by the BioGeomancer Consortium. In: Facility GBI, editor. Copenhagen2006. p. 1–77.

[64] Wieczorek J, Bloom D, Constable H, Fang J, Koo M, Spencer C, et al Georeferencing Quick Reference Guide. 2012.

[65] ESRI. ArcGIS Desktop: Release 10. Redlands, CA.2011. www.esri.com. Accessed 1 January 2013.

[66] OS. ESRI Shapefile, Boundary-Line of Cornwall. Southampton2017.

[67] Godefroid S, Dana ED. ORIGINAL ARTICLE: Can Ellenberg's indicator values for Mediterranean plants be used outside their region of definition? Journal of Biogeography. 2007;34(1):62–8. doi: 10.1111/j.1365-2699.2006.01582.x

[68] Bennie J, Hill MO, Baxter R, Huntley B. Influence of slope and aspect on long-term vegetation change in British chalk grasslands. Journal of Ecology. 2006;94(2):355–68. doi: 10.1111/j.1365-2745.2006.01104.x

[69] Curtis CA, Bradley BA. Plant Distribution Data Show Broader Climatic Limits than Expert-Based Climatic Tolerance Estimates. PloS one. 2016;11(11):e0166407 doi: 10.1371/journal.pone.0166407 27870859 10.1371/journal.pone.0166407 PMC5117642

[70] R Core Team. R: A language and environment for statistical computing. R Foundation for Statistical Computing Vienna, Austria 2016.

[71] Newbold T. Applications and limitations of museum data for conservation and ecology, with particular attention to species distribution models. Progress in Physical Geography. 2010;34(1):3–22. doi: 10.1177/0309133309355630

[72] Tsutsui ND, Suarez AV. The Value of Museum Collections for Research and Society. BioScience. 2004; 67:66–74.

[73] Baker DJ, Hartley AJ, Pearce-Higgins JW, Jones RG, Willis SG. Neglected issues in using weather and climate information in ecology and biogeography. Diversity and Distributions. 2017;23(3):329–40. doi: 10.1111/ddi.12527

[74] Elith J, Leathwick J. Predicting species distributions from museum and herbarium records using multiresponse models fitted with multivariate adaptive regression splines. Diversity and Distributions. 2007;13(3):265–75. doi: 10.1111/j.1472-4642.2007.00340.x

[75] Beaman RS, Conn BJ. Automated geoparsing and georeferencing of Malesian collection locality data. Telopea 2003; 10(1):43–52.

[76] Beaman R, Wieczorek J, Blum S. Determining Space from Place for Natural History Collections. In a Distributed Digital Library Environment. 2004;10(5).

[77] Isaac NJB, Pocock MJO. Bias and information in biological records. Biological Journal of the Linnean Society. 2015;115(3):522–31. doi: 10.1111/bij.12532

[78] Mair L, Ruete A. Explaining Spatial Variation in the Recording Effort of Citizen Science Data across Multiple Taxa. PloS one. 2016;11(1):e0147796 doi: 10.1371/journal.pone.0147796 26820846 10.1371/journal.pone.0147796 PMC4731209

[79] Bellamy C, Altringham J. Predicting Species Distributions Using Record Centre Data: Multi-Scale Modelling of Habitat Suitability for Bat Roosts. PloS one. 2015;10(6):e0128440 doi: 10.1371/journal.pone.0128440. PMC4460044. 26053548 10.1371/journal.pone.0128440 PMC4460044

[80] Boakes EH, McGowan PJK, Fuller RA, Chang-qing D, Clark NE, O'Connor K, et al Distorted Views of Biodiversity: Spatial and Temporal Bias in Species Occurrence Data. PLoS biology. 2010;8(6):e1000385 doi: 10.1371/journal.pbio.1000385 20532234 10.1371/journal.pbio.1000385 PMC2879389

[81] Boakes EH, Fuller RA, McGowan PJK, Mace GM. Uncertainty in identifying local extinctions: the distribution of missing data and its effects on biodiversity measures. Biology Letters. 2016;12(3). doi: 10.1098/rsbl.2015.0824 26961894 10.1098/rsbl.2015.0824 PMC4843216

[82] Tingley MW, Beissinger SR. Detecting range shifts from historical species occurrences: new perspectives on old data. Trends in Ecology & Evolution. 2009;24(11):625–33. 19683829 10.1016/j.tree.2009.05.009

[83] Filibeck G, Adams J, Brunetti M, Di Filippo A, Rosati L, Scoppola A, et al Tree ring ecological signal is consistent with floristic composition and plant indicator values in Mediterranean Fagus sylvatica forests. Journal of Ecology. 2015;103(6):1580–93. doi: 10.1111/1365-2745.12478

[84] Scherrer D, Körner C. Topographically controlled thermal-habitat differentiation buffers alpine plant diversity against climate warming. Journal of Biogeography. 2011;38(2):406–16. doi: 10.1111/j.1365-2699.2010.02407.x

[85] Kosanic A, Harrison S, Anderson K, Kavcic I. Present and historical climate variability in South West England. Climatic Change. 2014:1–17. doi: 10.1007/s10584-014-1101-8

[86] Ashcroft MB, Gollan JR, Warton DI, Ramp D. A novel approach to quantify and locate potential microrefugia using topoclimate, climate stability, and isolation from the matrix. Global Change Biology. 2012;18(6):1866–79. doi: 10.1111/j.1365-2486.2012.02661.x

[87] Ashcroft MB, Chisholm LA, French KO. Climate change at the landscape scale: predicting fine-grained spatial heterogeneity in warming and potential refugia for vegetation. Global Change Biology. 2009;15(3):656–67. doi: 10.1111/j.1365-2486.2008.01762.x

[88] Colles A, Liow LH, Prinzing A. Are specialists at risk under environmental change? Neoecological, paleoecological and phylogenetic approaches. Ecology Letters. 2009;12(8):849–63. doi: 10.1111/j.1461-0248.2009.01336.x 19580588 10.1111/j.1461-0248.2009.01336.x PMC2730552

[89] Wesche K, Krause B, Culmsee H, Leuschner C. Fifty years of change in Central European grassland vegetation: Large losses in species richness and animal-pollinated plants. Biological Conservation. 2012;150(1):76–85.

[90] Casalegno S, Bennie JJ, Inger R, Gaston KJ. Regional Scale Prioritisation for Key Ecosystem Services, Renewable Energy Production and Urban Development. PloS one. 2014;9(9):e107822 doi: 10.1371/journal.pone.0107822 25250775 10.1371/journal.pone.0107822 PMC4175084

[91] Suggitt A, Wilson R, August T, Fox R, Isaac NB, Macgregor N, et al Microclimate affects landscape level persistence in the British Lepidoptera. Journal of Insect Conservation. 2015;19(2):237–53. doi: 10.1007/s10841-014-9749-y

[92] Carpenter SR, Mooney HA, Agard J, Capistrano D, DeFries RS, Díaz S, et al Science for managing ecosystem services: Beyond the Millennium Ecosystem Assessment. Proceedings of the National Academy of Sciences. 2009;106(5):1305–12. doi: 10.1073/pnas.0808772106 19179280 10.1073/pnas.0808772106 PMC2635788

[93] Seddon N, Mace GM, Naeem S, Tobias JA, Pigot AL, Cavanagh R, et al Biodiversity in the Anthropocene: prospects and policy. Proceedings of the Royal Society B: Biological Sciences. 2016;283(1844). doi: 10.1098/rspb.2016.2094 27928040 10.1098/rspb.2016.2094 PMC5204156

