# Supplementary material for: Changes in the geographical distribution of plant species and climatic variables on the West Cornwall peninsula (South West UK)
Source: PLoS One. 2018 Feb 5;13(2):e0191021. doi: 10.1371/journal.pone.0191021 (PMC5798772; doi:10.1371/journal.pone.0191021)
Supplement: S1 Table — Showing: plant species (ID), Area that was lost (%), Classified climate indicator values (CV), Ellenberg values (EV), Mean climatic data for the plant species (Raw CV). (PDF) [file pone.0191021.s001.pdf]

ID	Area Lost (%)	CV			EV			Raw CV		
		Tjan	Tjul	RR	L	M	N	Jan	Jul	Precip
2	20.08	8	7	4	4	5	6	5.5	16.2	962
4	1.51	3	3	6	7	7	3	3.3	14.3	1146
5	14.37	4	4	4	8	3	5	3.6	14.9	986
6	23.06	4	4	5	4	6	7	3.5	14.8	1065
7	100.00	2	3	6	8	6	3	2.9	14.1	1185
8	0.00	8	6	5	8	4	5	5.5	15.5	1068
9	0.00	4	4	5	7	5	7	3.5	14.7	1032
10	21.58	5	7	3	9	4	3	4.1	16.1	804
12	84.98	5	5	4	7	4	5	4	15.2	962
13	2.86	4	6	2	7	4	6	3.7	15.6	797
14	11.85	4	6	3	7	5	6	3.8	15.7	809
20	14.95	4	7	2	8	3	4	3.8	16	753
21	38.25	3	3	6	7	4	2	3.2	14.4	1104
22	44.41	4	5	3	7	4	7	3.5	15.4	832
23	56.45	2	1	8	8	9	2	2.7	13.2	1404
24	9.05	3	3	7	7	6	2	3.3	14	1243
25	0.00	5	5	5	8	6	5	4.3	15	1039
26	14.10	6	4	7	8	7	5	4.5	14.5	1228
28	0.73	4	4	5	4	8	6	3.7	14.8	1063
29	1.09	4	5	4	5	5	5	3.6	15.2	950
30	24.83	5	7	2	7	4	3	4	16.1	744
31	0.00	4	5	3	7	5	5	3.8	15.4	836
33	12.41	5	7	3	8	8	3	4.4	16.1	841
34	4.77	4	5	4	6	5	7	3.7	15.3	905
35	0.00	5	7	2	8	6	7	4.2	16	797
36	0.00	4	7	2	7	6	8	3.9	16	794
37	20.89	5	7	2	7	5	7	4	16.1	762
38	44.07	5	7	2	7	4	9	4.1	16.1	752
41	79.81	4	6	2	8	2	1	3.6	15.8	778
42	11.05	4	5	4	8	5	8	3.9	15.2	915
43	0.00	4	7	2	8	5	6	3.7	16.3	711
44	0.00	5	6	3	7	6	2	4.3	15.9	846
45	18.92	8	7	4	8	9	5	5.7	16.1	943
46	69.04	2	2	8	6	7	4	2.6	13.5	1361
47	0.00	4	7	2	4	5	5	3.7	16	760
48	0.00	3	3	6	6	5	3	3.2	14.1	1177
50	0.00	4	6	3	8	7	7	3.8	15.6	863
51	75.85	3	1	8	8	9	1	3.2	13.4	1422
52	4.62	4	6	3	8	4	4	3.8	15.6	822
53	0.00	4	5	4	7	8	7	3.8	15	971
54	0.00	5	7	4	7	5	5	4.4	16	937
55	0.00	4	6	3	6	7	5	3.9	15.9	834
56	10.02	4	3	6	8	10	4	3.5	14.4	1116
57	0.00	3	3	7	8	8	1	3.4	14	1230
58	100.00	6	7	3	9	3	3	4.6	16.1	856
59	18.32	5	7	3	4	5	6	4.1	16.1	827
60	11.74	4	6	2	6	4	5	3.9	15.8	783
61	0.00	4	6	3	6	5	5	3.8	15.8	804

62	100.00	8	7	4	9	4	5	5.8	16.1	969
63	13.65	7	5	5	8	3	3	5.3	15.4	1060
64	100.00	5	5	3	8	4	3	4.2	15.4	828
65	0.00	5	6	3	9	5	5	4.1	15.9	823
66	0.00	5	4	4	7	4	7	4.2	14.9	997
67	43.34	3	4	5	8	5	2	3	14.6	1019
68	0.00	4	5	4	6	4	6	3.6	15.1	991
69	0.00	5	7	3	7	4	6	4.2	16.2	810
72	0.00	8	7	3	9	4	1	6.5	16.2	887
73	4.17	5	6	3	5	7	5	4	15.6	884
74	0.00	5	7	3	5	4	5	4.2	16.1	821
75	0.00	4	5	4	7	7	5	3.8	15.2	925
76	39.10	6	5	5	8	8	5	4.7	15.1	1081
77	64.35	2	1	8	8	5	3	2.8	13.4	1380
79	0.00	3	3	6	6	5	3	3.3	14.2	1172
80	0.00	4	7	2	8	6	6	3.9	16.3	742
81	52.20	7	7	3	9	4	5	5.2	16.1	875
82	0.00	4	7	2	7	4	4	3.7	16.1	717
83	0.00	5	5	5	6	5	6	4.3	15.4	1027
84	0.00	4	6	3	6	5	5	3.7	15.7	865
85	23.42	4	6	3	7	4	5	3.8	15.7	804
86	100.00	4	6	2	8	4	5	3.7	15.9	755
87	6.11	5	6	3	7	4	7	4	15.6	855
88	22.48	5	7	3	4	4	2	4.2	16.2	859
89	79.56	4	4	6	9	9	1	3.5	14.9	1122
90	0.00	1	1	8	7	5	2	2.4	13.3	1368
91	0.00	4	5	4	7	9	5	3.7	15.2	943
92	0.00	4	6	3	7	3	4	3.7	15.6	871
93	15.53	6	7	3	9	5	8	4.6	16.1	861
94	65.89	4	7	1	8	3	3	3.5	16.2	604
96	9.62	5	6	3	8	7	7	4.4	15.9	869
97	4.04	5	7	3	6	4	6	4.4	16.1	861
98	0.00	8	8	2	9	3	2	6.8	16.6	718
99	37.25	5	7	3	7	7	3	4.4	16.2	849
100	10.55	4	6	2	9	4	4	3.9	15.9	777
101	37.63	4	6	3	8	4	3	3.9	15.8	801
102	0.00	4	5	3	6	4	4	3.7	15.3	898
103	0.00	4	6	4	7	4	3	3.9	15.5	935
104	0.00	5	7	2	7	4	6	4	16.1	774
105	0.00	4	6	3	7	4	5	3.9	15.8	843
106	22.83	4	6	2	7	4	5	3.7	15.7	759
107	0.00	4	5	3	7	4	8	3.7	15.3	868
108	0.00	8	7	4	9	4	4	6.3	16.3	923
109	36.44	5	8	1	8	8	6	4.3	16.6	661
111	8.79	3	4	6	6	5	6	3.3	14.5	1102
112	0.00	5	4	5	8	7	2	4.2	14.7	1061
113	0.61	4	6	2	7	5	6	3.7	15.9	742
114	19.65	7	6	5	9	10	3	5.2	15.8	1024
115	33.71	4	3	8	8	9	1	3.6	14	1400
116	5.94	4	4	4	9	4	3	3.7	14.9	989

117	0.00	4	2	7	8	6	4	3.7	13.9	1291
118	11.37	4	3	7	8	7	3	3.5	14	1205
119	0.00	4	4	5	7	8	6	3.7	14.8	1030
120	43.61	8	7	4	9	3	1	5.9	16.2	939
121	0.00	4	4	4	7	5	6	3.6	14.9	987
122	16.50	4	6	2	7	4	7	3.9	15.9	798
124	0.00	4	4	4	8	3	2	3.5	14.9	983
125	2.18	3	3	6	7	4	3	3.4	14	1185
126	0.00	8	7	3	9	4	2	6.5	16.2	867
127	100.00	6	7	2	9	3	2	4.6	16.3	767
128	26.45	6	8	2	9	6	6	4.5	16.5	757
129	23.54	4	5	4	7	4	4	3.6	15.2	900
130	0.00	4	5	4	8	5	8	3.7	15	914
131	0.00	3	4	6	6	8	5	3.4	14.5	1115
132	8.04	4	7	2	7	4	6	3.9	16.2	742
133	0.00	4	5	4	7	4	5	3.7	15.2	918
134	0.00	4	4	5	4	6	6	3.6	14.9	1029
135	0.00	5	7	3	7	4	4	4.4	16.1	827
136	0.00	7	6	5	7	6	2	5	15.8	1025
137	0.00	4	6	3	5	5	7	3.8	15.7	833
138	0.00	5	5	4	7	12	5	4.3	15	926
95	100.00	1	1	8	4	5	3	1.9	13	1483
